# Genital Tract GAS Infection ISIDOG Guidelines

**DOI:** 10.3390/jcm10092043

**Published:** 2021-05-10

**Authors:** Gilbert Donders, Peter Greenhouse, Francesca Donders, Ulrike Engel, Jorma Paavonen, Werner Mendling

**Affiliations:** 1 Femicare, Clinical Research for Women, 3300 Tienen, Belgium; francesca.donders@gmail.com; 2 Department of Obstetrics and Gynecology, University Hospital Antwerp, 2000 Antwerp, Belgium; 3 Regional Hospital H Hart, 3300 Tienen, Belgium; 4 Clifton Women’s Health, Bristol BS6 6JD, UK; peter.gh@icloud.com; 5 Department of Ob/Gyn Maternité, Centre Hospitalier, 1210 Luxembourg, Luxembourg; ulrike.engel@me.com; 6 Department of Obstetrics and Gynecology, Helsinki University Hospital, 00260 Helsinki, Finland; jorma.paavonen@helsinki.fi; 7 German Centre for Infections in Gynaecology and Obstetrics, St. Anna Hospital, 42109 Wuppertal, Germany; w.mendling@t-online.de

**Keywords:** *Streptococcus pyogenes*, sepsis, puerperal infection, maternal mortality, invasive GAS infection, endometritis

## Abstract

There has been an increasing worldwide incidence of invasive group A streptococcal (GAS) disease in pregnancy and in the puerperal period over the past 30 years. Postpartum Group A streptococci infection, and in particular streptococcal toxic shock syndrome (TSS) and necrotizing fasciitis, can be life threatening and difficult to treat. Despite antibiotics and supportive therapy, and in some cases advanced extensive surgery, mortality associated with invasive group A streptococcal postpartum endometritis, necrotizing fasciitis, and toxic shock syndrome remains high, up to 40% of postpartum septic deaths. It now accounts for more than 75,000 deaths worldwide every year. Postpartum women have a 20-fold increased incidence of GAS disease compared to non-pregnant women. Despite the high incidence, many invasive GAS infections are not diagnosed in a timely manner, resulting in potentially preventable maternal and neonatal deaths. In this paper the specific characteristics of GAS infection in the field of Ob/Gyn are brought to our attention, resulting in guidelines to improve our awareness, early recognition and timely treatment of the disease. New European prevalence data of vaginal GAS colonization are presented, alongside two original case histories. Additionally, aerobic vaginitis is proposed as a supplementary risk factor for invasive GAS diseases.

## 1. Introduction

Group A streptococci (GAS), or *Streptococcus pyogenes*, is a widely carried pathogen, sometimes asymptomatically, or causing mild localized self-limiting infections such as impetigo and pharyngitis. Yet invasive GAS (iGAS) causes rare but serious infectious conditions in women: Toxic Shock-Like Syndrome (TSLS) which is associated with early onset shock and multi-organ system failure, necrotizing fasciitis (NF) which involves local necrosis of subcutaneous soft tissues and skin, and bacteremia which can cause focal infections including meningitis, pneumonia, peritonitis, osteomyelitis, septic arthritis, myositis, postoperative wound infections and puerperal sepsis, the latter being a major cause of maternal mortality. Incidence of these invasive infections is very low, around 1–8 per 100,000 population per year, but has a potential case fatality rate ranging from 5% to 20%, and in the case of septic shock up to 30–45% [1,2,3,4]. One out of 50 iGAS cases concerns a postpartum patient, with a case-fatality rate of 3.5% [5].

Many of those involved in women’s healthcare, including gynecologists and midwives, have little or no experience of recognizing and/or managing these conditions. Therefore, it is important to raise awareness and to have accessible guidelines to help to prevent and manage severe GAS disease. As there are no randomized trials studying invasive GAS disease, we agreed that a narrative review, demonstrative case report discussions, and internal consensus was the best way to inform and stimulate health care workers to prevent iGAS cases. Still, where possible, the grading of evidence of the consensus statements were added, based on the Oxford Centre for Evidence-Based Medicine: Levels of Evidence (March 2019) accessible on: https://www.cebm.ox.ac.uk/resources/levels-of-evidence/oxford-centre-for-evidence-based-medicine-levels-of-evidence-march-2009, (accessed on 14 March 2021). In collating the prevalence data across Europe, ethical committee approval was discussed in each country and approval was obtained where this was required.

A critical point linked to the patient’s mortality in most cases is a delay in diagnosis and subsequently belated therapeutic action. Failure to identify a discernable site of bacterial entry or a clear clue in the patient’s presenting history often leads to late recognition of the disease and its imminent dangers. Before anyone realizes what is going on, this delay results in inadequately managed systemic shock and multiple organ failure, with subsequent high incidence of morbidity and mortality. Early recognition and timely action in this rare, but lethal disease will be the cardinal features of these guidelines. The International Society for Infectious Diseases in Obstetrics and Gynecology (ISIDOG) provides a scientific forum for any health care worker who is involved in the care of women, and provides recommendations on less common infections which are often not covered by other societies’ guidelines.

### 1.1. GAS Bacteriology

*Streptococcus pyogenes* is a beta-hemolytic bacterium belonging to Lancefield serogroup A, also known as the group A streptococci (GAS). There are many different serotypes of *S. pyogenes*, which are non-sporing, non-motile cocci, each possessing a range of pyrogenic exotoxins which contribute to their ability to invade tissue and create systemic shock.

The earlier classification system was based on the surface antigen “M protein” (serotyping), a key virulence determinant, and the principal target for host phagocytes. Different genotypes are now characterized by over 200 variations in the emm gene, which theoretically can be used to identify and predict phenotypically-specific virulence [6], linked to several nucleotide polymorphisms in an intergenic region which significantly alters global transcript profiles affecting virulence [7]. Up to now, however, no schemes based on such genotypes are used in routine practice to predict virulence.

### 1.2. Pathogeniticy

#### 1.2.1. Bacterial Characteristics

Genomic and molecular analysis has identified several determinants of GAS virulence affecting adhesion, colonization, innate immune resistance, tissue barrier degradation and invasive spread (Table 1). GAS infection also triggers serious post-infectious immune-mediated disorders, including acute rheumatic fever and rheumatic heart disease. The principal virulence factors of GAS are attachment of the organisms and subsequent spread through infiltration and breakdown of connective tissue layers, while evading and suppressing host immune defences [8].

Minor virulence factors of different GAS strains are also determined by disparate plasminogen activation of SK subtypes, coupled with the strain’s specific M protein [9], although the clinical relevance of this is not yet known. Detailed information about individual components of GAS virulence is reviewed in: Streptococcus pyogenes: Basic Biology to Clinical Manifestations. Ferretti JJ, Stevens DJ, Fischetti VA (Eds) https://www.ncbi.nlm.nih.gov/books/NBK333424/, (accessed on 14 March 2021).

Recent work has focused on the biofilm in which *S. pyogenes* persists and could potentially resist host immunity [10]. Each moist mucous membrane is generally covered by a bacterial biofilm, which is a community of organisms encased in a matrix of extra-polymeric substances protecting them from hostile influences, including desiccation, antibiotics, other pathogens, and the host immune response. However, ablation of the biofilm may further contribute to GAS dissemination, the effect being controlled by various transcription regulators and response genes [11]. According to some, biofilms only seem to occur in laboratory settings, not playing an important role in in vivo, but according to others, bacteria also aggregate in vivo [10].

In order to find clues to better understand the pathogenesis of iGAS, Zhu et al. used transposon-directed insertion-site sequencing (TraDIS) to study the virulence gene expression of 2 specific GAS serotypes known to be involved in necrotizing myositis in humans [12,13]. They discovered that only a limited number of transporter genes (importers of amino-acids and carbohydrates, as well as exporters for toxins and quorum-sensing peptides) were responsible for the fitness of the two pathogenic strains, which also contributed to the development of myositis in non-human primates. Through deletion of specific genes which both bacteria had in common, they were able to single out five transporter genes that were strongly involved in the pathogenesis of this complication. They furthermore discovered that in specific clinical presentations, such as myositis, pharyngitis and puerperal sepsis, a different set of genes is involved. As we cannot alter the intrinsic characteristics of the bacterium affecting virulence and pathogenicity, we should focus on prevention rather than treatment of imminent invasive GAS disease. Development of vaccines based on the knowledge of the TraDIS experiments and timely use of penicillin are crucial in this respect.

#### 1.2.2. Host Response

Human immune responses against *S. pyogenes* consist of a robust Th1 cellular memory response in combination with IgG1/IgG3-dominated humoral immunity which increase with age. This makes children and young adults more vulnerable to invasive disease [14], especially during pregnancy.

### 1.3. Pathology–General

#### 1.3.1. Presentation and Pathology of Invasive Infection

The principal features of invasive GAS infections are an initial seemingly benign area of inflammation of skin or mucous membrane, minor surgical trauma, or mild soft tissue injury, followed by deeper necrosis (necrotizing fasciitis) or abscess formation in muscle (myositis), swiftly followed by shock and multi-organ failure. This covert presentation often gives little warning of the severity of underlying disease, similar to the course seen in severe staphylococcal toxic shock syndrome [15]. Recently, specific *S. pyogenes* types M1 & M3 are increasingly more likely to be associated with life-threatening infections [16]. Of importance, increased prevalence of macrolide-resistant GAS (MRGAS) and fluoro-quinolone resistant strains have emerged, requiring close surveillance [17] First discovered in the mid-1980s, the hypervirulent M1T1 strain is now rapidly spreading around the globe [18]. In depth studies of the evolutionary selection and pressure of such pandemic strains can help to develop new strategies to prevent the dissemination of future clones.

#### 1.3.2. Necrotizing Fasciitis

Necrotizing fasciitis (NF, or Streptococcal Gangrene) is an infection of the deeper subcutaneous tissues and fascia characterized by extensive and rapidly spreading necrosis of the skin and underlying structures [19]. The original minor lesion develops rapidly over some 1–3 days: mild skin erythema becomes more extensive, swollen, and florid, darkening to a purple color with ecchymoses and bullae containing yellow serous or hemorrhagic fluid appearing by 2–3 days. Unseen beneath the skin surface, deep muscle necrosis with destruction of soft tissue layers progresses due to a direct bacterial toxin effect on the arterioles and surrounding tissues, leading within 4–5 days to overt gangrene and skin sloughing which continues unless contained by timely antibiotics and aggressive surgery. Associated bacteremia leads to metastatic abscess formation, by which time the patient is severely systemically ill with high pyrexia and hypotensive shock. Despite appropriate treatment, mortality rates are globally high at around 70–80% [20].

Early recognition of NF, the key to successful treatment, is hampered by an initial benign cutaneous presentation, or in some patients by deep covert infective fasciitis at a point of trauma such as a hematoma, muscle strain or joint injury (with, in up to 50% of cases, no clear route of entry). While X-Ray, CT and MRI may reveal localized deep swelling, they may not show frank abscess formation or gas in tissue spaces, making interpretation difficult, thus potentially hampering early accurate diagnosis. The most important early clinical clues of hidden NF are fever associated with severe crescendo pain [21], which may both be masked by painkillers taken after any original trauma or surgery.

Other warning signs of NF in addition to pain and pyrexia are unexplained tachycardia, a marked neutrophil left shift, and raised creatine phosphokinase level, prompting an early biopsy or diagnostic aspiration of fluid—this will show Gram-positive cocci with very few white blood cells.

#### 1.3.3. Myositis and Myo-Necrosis

Myositis is a localized infection of muscle with a purulent central area leading to formation of an abscess, of which *S. pyogenes* is a rare cause. Myco-necrosis is a more common non-purulent soft tissue infection: both occur at sites of blunt trauma, or arise spontaneously in soft tissue, derived from hematogenous spread from the throat. Both symptoms and the underlying pathological process overlap with NF, leading rapidly to systemic shock, with similarly high (~80%) mortality [22].

#### 1.3.4. Bacteremia

Despite *S. pyogenes* bacteremia being rare due to low-threshold use of antibiotics, increases have been seen in healthy adults between 20 and 50 years old, in IV drug users and people residing in nursing homes. Malignancy, immune depression, diabetes mellitus and peripheral vascular disease are predisposing factors in older adults, and, as in children with skin lesions such as varicella, the portal of entry is usually the skin. Its course can be benign, but often starts suddenly, with chills, high fever, prostration, and high mortality in the range of 15% to 38% [23,24,25]. In a more recent survey, smoking, alcohol consumption, and chronic lung diseases were also mentioned as risk factors, and there was a clear and consistent seasonal variation, with increased incidence rates of iGAS in winter and spring versus summer and autumn [4].

#### 1.3.5. Streptococcal Toxic Shock Syndrome

Streptococcal Toxic Shock Syndrome (StrepTSS) is any streptococcal infection associated with sudden onset shock and organ failure, diagnosed definitively by isolation of *S. pyogenes* from a normally sterile body site. StrepTSS develops from *S. pyogenes* spread from the vagina, pharynx, mucosa, or skin. It can occur after hysterectomy, vaginal or caesarean delivery, and occasionally after systemic viral infections such as varicella and influenza, but only rarely after streptococcal pharyngitis [26]. The condition is mostly sporadic, with rare clusters seen in nursing homes and hospitals. Transmission can occasionally occur to family members or health care workers [27].

Special vigilance is needed to prevent community-acquired strepTTS (e.g., in nursing homes). One single case of strepTTS infected up to 23 hospital workers with *S. pyogenes* [28]. The incidence of invasive *S. pyogenes* infections in the United States is 3.5/100,000 population/year, but the risk to contacts is estimated to be 20 times greater. Still, this remains a rare disease, as according to CDC calculations, around 1500 contacts with a case with invasive TTS are needed to cause one secondary invasive case (Prevention of Invasive Group A Streptococcal Infections Workshop Participants, 2002, Centers of Disease Control, Atlanta).

Originally seen mainly in the young, the aged or infirm, nowadays people of any age can be affected: Diabetes and alcoholism are common precursors, but many cases have neither predisposing medical conditions nor are they immune-compromised. StrepTTS is diagnosed by the combination of hypotension and signs of multi-organ failure (Table 2).

Given the relative infrequency of these infections and the lack of a clearly effective chemo-prophylactic regimen, routine screening for and prophylaxis against streptococcal infection are not recommended for household contacts of index patients. In deciding who should receive prophylaxis, the clinician needs to consider the duration and intimacy of contact and underlying host factors of individual contacts. However, contacts with open wounds, persons after surgery or childbirth, or patients who have concurrent viral infections such as varicella or influenza or immunodeficiency should receive prophylaxis with penicillin, clindamycin, or azithromycin (Prevention of Invasive Group A Streptococcal Infections Workshop Participants, 2002).

#### 1.3.6. Long Term Sequelae

After acute infection, several long-term sequelae can ensue, such as acute rheumatic fever, rheumatic heart disease and glomerulonephritis. Worldwide, it is estimated that severe GAS disease (including these complications) is responsible for over 500,000 deaths annually [29]. As these complications are not within the scope of this paper, they will not be further discussed here.

### 1.4. Pathology–Pregnancy

The discovery by Semmelweis in 1850 that insufficient hand hygiene (later discovered to be due to nosocomial infection with *S. pyogenes*) caused an excess of maternal mortality in a hospital in Budapest cost him his job and finally his life. Nowadays nosocomial infection only constitutes a minority (15%) of the causes of puerperal sepsis in Western countries, but the paramount importance of preventing nosocomial infection should still be emphasized. Another 20% occurs during the third trimester, prior to the onset of labor or rupture of membranes [30].

Fever, abdominal pain, and hypotension are frequent signs of imminent sepsis, but absence of tachycardia, sometimes hypothermia and typically absence of leukocytosis can make the diagnosis tricky (Case 1). In the US, an overall rate of six cases of pregnancy-related GAS sepsis per 100,000 live births occurs, with a case fatality rate of 3.5 % [5]. Risk factors for maternal mortality are the later third trimester or early postpartum (<4 days) period [30].

#### 1.4.1. Case 1

A pregnant woman G2P1 presented at term to the delivery ward of a tertiary hospital with contractions starting. Her first pregnancy and delivery 3 years previously was uneventful. She had a red, swollen vulva with excessive yellow brownish vaginal discharge and was prescribed vaginal miconazole for vulvitis. Rectovaginal specific culture for detecting group B streptococci (GBS) taken at 35 weeks was negative. After an uneventful labor in a water bath, the patient delivered a healthy girl of 3.35 kg the same day.

During the first postpartum days she progressively developed complaints of fatigue and muscle pains. On day 3 a point of care test for flu was negative, leading to a presumed diagnosis of psychic decompensation and onset of postpartum depression. The nurses’ notes record that the patient was then in severe pain, could not move out of her bed, and was not able to take care of her newborn child, which was interpreted as catharsis due to severe depression. C-reactive protein (CRP) increased, while leukocytosis was low (3300 Leukocytes per mL). Temperature was 36.5 and blood pressure and heart rate normal. Thrombocytes were 123 × 10^9^/mL on day 3. By day 4 thrombocytes had dropped to 64 × 10^9^/mL and petechiae became visible on the skin of her arms and legs. An internist was called in, and urine and blood cultures were ordered. Pain killers and antidepressants had been started, but not antibiotics. Twenty-four hours later, blood cultures came back positive for *S. pyogenes*, and intravenous ampicillin was started, but the patient died that night.

#### 1.4.2. Learning Points

1. Although asymptomatic carriage of GAS is usually encountered in the upper respiratory tract, most often in children (15% to 20%) [31], it can also occur rarely in the vagina of 0.03% to 0.3% of asymptomatic women [32]. Often a child in the family colonized with GAS is the index carrier.
2. A negative GBS test at 35 weeks is not a proof of absence of GAS and awareness of GAS has to be high.
3. The type of vaginitis in this case should have prompted proper diagnostic workup including microscopy and culture of the vaginal discharge, instead of simply prescribing antimycotics. This would most likely have detected the GAS in time, and antibiotics at that point would without doubt have saved this woman. Invasive postpartum GAS disease after aerobic vaginitis due to GAS has been reported [33]
4. Invasive GAS is 20 to 100 times more frequent in the peripartum period (late third trimester and especially first postpartum days) and occurs more often in already colonized women, as was most likely the case here [34].
5. Signs of severe muscle pain often accompanied by abdominal pain should prompt the presumptive diagnosis of (pre-)sepsis. Absence of tachycardia, high fever or leukocytosis in this case can all have contributed to the fact that the diagnosis of iGAS came too late, and to why other diagnoses such as ’flu and depression were considered instead [2]. Unexplained tachycardia without fever should also prompt consideration of the diagnosis [35].
6. Increased awareness and timely initiation of antibiotics (before culture results are known) save lives in cases of GAS infection, especially in the puerperal period.

### 1.5. Epidemiology

#### 1.5.1. General Prevalence in Women

GAS is present on the skin or in the throat of 5–30% of the population and is easily spread by air droplets or direct person-to-person contact [31].

In routine surveillance of scarlet fever cases in the UK, there is a clear seasonal variation with a significant annual peak around March, and with a recent year-on-year increase [32]. It is not known whether this reflects genital carriage of the organism or is relevant for the prediction of invasive GAS disease in obstetrics or gynecology.

#### 1.5.2. Prevalence of Colonization of the Genital Tract

In comparison with high colonization on skin and in the throat, the presence of GAS in the vagina is relatively rare (0.03%) and colonization per se is not sufficient to cause disease [36]. We performed a literature search and questioned the ISIDOG country managers about the prevalence of *S. pyogenes* in their region. Table 3 shows the 10-year lab-based culture results of the hospitals in which ISIDOG members were working. There is a prevalence of 0.1 to 2.4%, but clearly in countries where cultures were only taken in symptomatic patients’ prevalence was higher (Sweden 2.2%, The Netherlands 2.4%), than in countries where routine and control checks were tested (Switzerland 0.1%, Belgium 0.2%, Germany 0.4%, *p* < 0.0001).

In the published literature, as summarized in Table 4, GAS carriage in healthy, asymptomatic women (Table 4, part a) is also much lower than in women with signs or symptoms of vaginitis (Table 4, part b). These data clearly show that prevalence of GAS is much higher in women with symptoms of vaginitis [37,38,39,40,41,42,43]. GAS vaginitis is mostly found in pre-pubertal girls and postmenopausal women due to a thin un-estrogenized vaginal epithelium which is more vulnerable to inflammation and infection. Based on microbiological studies, GAS is the most common cause of vulvitis or vulvovaginitis in pre-pubertal females. On the other hand, GAS vaginitis is rarely found in adult, premenopausal women.

In New Zealand, with a general vaginal GAS prevalence among almost 5000 women of 2%, and 1% in hospital patients (*n* = 2000), more symptomatic women (mainly with discharge) were encountered in the community, while hospitalized patients were more likely to have ascending infection related to pregnancy [32].

There are some case reports which present with peritonitis due to ascending vaginal infection caused by GAS in the menopause. What is almost never reported on is the risk of invasive GAS as a result of invasive infertility procedures. Here we present a case of peritonitis in a woman presenting with infertility (Case 2).

##### Case 2

A 29-year-old nulligravid woman, without relevant previous history, presented for in vitro fertilization and her first transvaginal ovarian pick-up procedure after hormonal ovarian hyperstimulation. During the procedure, the bladder was evacuated using a disposable sterile catheter, and the vagina was rinsed with 0.9% NaCl. No disinfectant was used, due to potential ototoxic activity. The procedure was uneventful. Two days later she presented in the emergency room with progressive severe lower abdominal pain and dysuria. She had no fever at the time of admission and had normal vital signs. Clinically, abdominal tenderness, distension and mild peritoneal tenderness was found, but there was no costovertebral angle tenderness. On transvaginal ultrasound, enlarged ovaries and a small pocket of fluid in the Pouch of Douglas were seen, as expected after the stimulation and pick-up procedures. Laboratory work up showed hemoglobin of 12.7 g/dL, leukocytosis of 22,000/L and a C-reactive protein (CRP) of 95 mg/L. Kidney and liver function were normal. Urinary sediment was positive with 899 red blood cells/mL and >1000 white blood cells/mL. She was admitted to the ward with presumed ovarian hyperstimulation syndrome in combination with a complicated urinary infection and treated with oral furadantin 100 mg 3 times daily and 1 L intravenous (IV) crystalloid fluid therapy (restricted because of the assumed hyperstimulation syndrome).

Overnight she developed a pyrexia of 39 °C. Blood cultures were taken and a computed tomography scan (CT) with contrast was performed, showing no signs of abscess or bowel perforation. IV fluid therapy was increased to 2 L/day and preliminary results of the urine culture showed *S. pyogenes*, prompting switching antibiotic to g IV amoxicillin four times daily, awaiting further resistance profile results. Urinary output was normal at 1000 mL/24 h. Two days after admission the patient rapidly deteriorated clinically, leukocytosis increased to 34.000/mL and CRP to 490 mg/L, while kidney function decreased with a serum creatinine level of 1.4 mg/dL.

A diagnostic laparoscopy was performed, and a diffuse pelvic infection/peritonitis was diagnosed with purulent secretions throughout the abdomen. No focal abscesses were seen. Cultures were taken and thorough rinsing of the abdominal cavity was performed with 0.9% NaCl. Antibiotic treatment was switched to tazocin 4 g 3 times daily and a single dose of azithromycin. At post-op day 1 she developed hypovolemia, hypotension, and acute kidney failure with anuria (urinary output of less than 75 mL/24 h). She was admitted to the intensive care unit where IV fluids were combined with a loop diuretic drip (furosemide). Postrenal causes were excluded by ultrasound. All cultures of the abdominal cavity came back positive for *S. pyogenes*, while no other micro-organisms were detected, and PCR for Chlamydia was negative. Antibiotic treatment was switched to penicillin G IV 6 million units/day. The patient recovered slowly and was discharged from hospital 14 days after admission.

##### Learning Points

1. Seemingly harmless interventions can cause life threatening complications in GAS positive patients
2. Even in the absence of an antibiogram, immediate initiation of sufficient doses of a narrow spectrum penicillin is warranted
3. Awareness and rapid (presumptive) diagnosis and intervention is lifesaving

#### 1.5.3. Prevalence of iGAS in Pregnancy and Postpartum

Puerperal sepsis is still a major concern around the world as it causes more than 75,000 maternal deaths annually, mostly in developing countries, where puerperal infections affect at least 5% of pregnant women [44]. While today group B streptococci, gonococci, Chlamydia, herpes simplex, genital Mycoplasma, and bacterial vaginosis are more often implicated in postpartum fever than GAS, severe cases of puerperal infections are almost always caused by the latter.

It has been noted that the obstetric patient is especially vulnerable to invasive GAS infection acquired via disrupted mucosal or cutaneous barriers during delivery. Postpartum GAS infections in the modern era are a mixture of illnesses of endogenous (patient) origin and acquired (iatrogenic/nosocomial) infections. Outbreaks of postpartum GAS infection continue to be reported and are often related to the spread of GAS among postpartum patients by healthcare workers who are asymptomatic carriers [5]. For example, rectal colonization of an obstetrician was implicated in nine postpartum GAS infected cases [45] and vaginal colonization of an operating room nurse was suspected to be involved in 18 wound infections, often requiring major surgical intervention afterwards [46].

Despite low rates of GAS in rectovaginal swabs in late pregnancy (0.03%), GAS disease is 20-fold higher for pregnant and postpartum women compared with non-pregnant women. A 13-year retrospective cohort study in Israel noted an incidence of peripartum invasive GAS of 88 cases per 100,000 persons (0.09%), of whom 93% were postpartum [47].

In London and the South East of England between 2010 and 2016 the incidence of invasive GAS among women within 28 days postpartum was 0.1% compared with 0.001% in non-pregnant females aged 14–44 [34].

#### 1.5.4. Incidence of Mortality Due to GAS

While the incidence rate of GAS carriage in pregnancy may seem relatively low, invasive GAS infections are significant because of their relatively high maternal mortality rate, with 20% of women dying within 7 days of diagnosis [48]. Even more relevant to the obstetrician are the findings of the Saving Mothers’ Lives report, which highlights that GAS infection was the leading cause of death due to puerperal sepsis [49]. This report mentions 26 direct deaths from maternal sepsis (1.13 per 100,000 pregnancies) during the period 2006–2008, 13 (50%) of which were due to proven GAS infection. During the period 2014–2016, the rate of maternal sepsis decreased to 0.48 per 10,000 pregnancies. In Japan GAS was also the most common causative bacterium for maternal sepsis-related death in the period 2010–2014 (54%) [50].

### 1.6. Diagnosis

#### 1.6.1. Clinical Diagnosis

The most important clue for an adequate and timely diagnosis of iGAS is awareness of the disease and its subtle development in the early (and even late) phases. There are three levels of GAS complication: (1) ‘Mild GAS’, such as pharyngitis or impetigo, spread through direct contact with mucus from the nose or throat of an infectious person, (2) ‘Invasive GAS’ (iGAS), such as necrotizing fasciitis and toxic shock syndrome and (3) late immunologic complications like rheumatic fever, glomerulonephritis, and cardiac disease. Here we will only focus on early recognition of iGAS, as it affects women, and especially pregnant and postpartum women, at a disproportionate rate.

In a typical case blood pressure drops rapidly, the skin shows a rash, temperature peaks and patients are generally not well, confused and in severe distress. In the end, all vital organs such as kidney, liver, and lungs start to fail. Failure to recognize such symptoms may rapidly lead to death, even within a day.

The presence of risk factors should trigger the suspicion of potential iGAS infections. Due to the frequent harmless presence of GAS-like aerobic bacteria in the bowel or on the skin, the higher likelihood of GAS in women with symptoms of vaginitis, and its link to infection-related complications in pregnancy such as chorio-amnionitis, preterm labor and neonatal sepsis, the presence of aerobic vaginitis (AV) was added to that list (Table 5). Indeed, besides our case presented, there is further evidence in the literature that GAS positive AV can be the port of entry of iGAS, especially during the postpartum period [33]. Early recognition of pre-sepsis signs and immediate treatment are crucial and undoubtedly save precious lives (Table 6). Particularly, as demonstrated in case 1 above, the features of imminent severe sepsis should not be missed: a lethargic, confused patient with subfebrile or low temperatures, low blood pressure, and paradoxically low white blood cell count is likely to be more ill than often suspected, and this may evolve into a fatal outcome within hours (Table 6).

Diagnostic workup should include blood culture (prior to antibiotic treatment), blood count for leucocytes (which may be high or paradoxically low), serum lactate (lactate >4 mmol/L indicates tissue hypo-perfusion), chest X-Ray (mainly for puerperal sepsis), ultrasound scan of the uterus to detect retained placental products, and computed tomography scan (if pelvic abscess is suspected). X-rays of the affected area may show soft tissue gas in the later stages and may be useful in ruling out other septic conditions such as osteomyelitis. Computed tomography or magnetic resonance imaging of the affected area can help in assessing the extent of involvement and planning surgical debridement (in the case of necrotizing fasciitis). Doppler sonography is useful if thrombosis is suspected. Full blood count, urea, electrolytes, and C-reactive protein should be performed at regular intervals, e.g., every 6 h. Creatinine above 175 µmol/L (renal impairment), blood platelets < 100 × 10^9^/L (coagulopathy or disseminated intra vascular coagulation) and ALT/AST or bilirubin levels twice the normal upper limit for age (liver damage) may indicate imminent multi-organ failure. Oedema and hyperglycemia in the absence of diabetes (glucose > 7.7 mmol/L) are less well-known markers of imminent sepsis.

Women with symptoms of tonsillitis/pharyngitis should have a throat swab sent for culture. A pre-moistened nose swab may be sent for rapid MRSA screening, and throat swabs, midstream urine, high vaginal swab, rectal swabs, placental swabs, and sputum should be sent for culture (especially GAS). If indicated, cerebrospinal fluid and epidural site swabs can also be sent for culture. Rapid tests such as fresh wet mounts or Gram stain of cervico-vaginal smears showing (gram positive) cocci in chains or pairs, the ‘Rapid Strip Test’ or bedside PCR tests can dramatically enhance diagnostic efficacy by obtaining rapid results within hours or even minutes. Recently, molecular amplification assays targeting Mf (Methylene-tetra-hydro-folate reductase) genes and cfb (cyclic AMP factor of B streptococci) genes are able to differentiate between GAS and GBS, respectively.

#### 1.6.2. Confirmatory Bacteriology Techniques

##### Identification Techniques (Culture, Species Testing and Microscopy)

Sheep blood agar media enriched with catalase allows detection of β-hemolysis, and enhances the growth of streptococci. Apart from normal growth conditions, special procedures have been developed to optimize identification of *S. pyogenes* in throat cultures [59]. In most cases of acute streptococcal pharyngitis or iGAS, ample growth of typical colonies can be observed after 24 h of incubation at 35–37 °C. If only a few colonies of *S. pyogenes,* collected from the patient’s pharynx, appear after incubation under these conditions, interpretation becomes more difficult, and these patients are most likely to be streptococcal carriers [60].

Identification of *S. pyogenes* requires bacitracin susceptibility and PYR activity testing, followed by the definitive Lancefield group A antigen test. It takes 48 h to confirm negative results.

**Figure 1:**
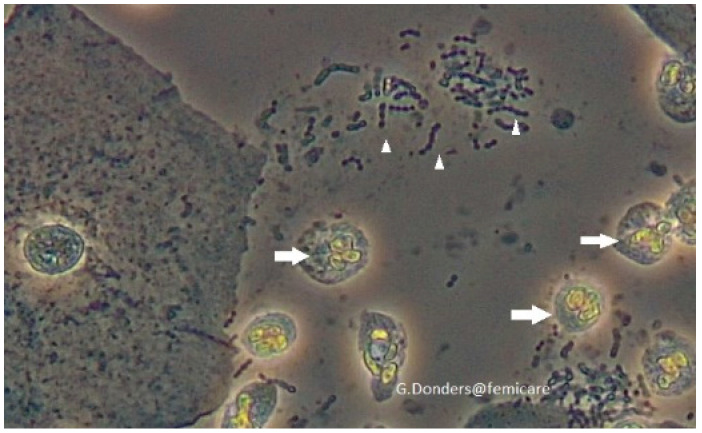
Bedside fresh wet mount microscopy of *S. viridians* in vaginal fluid. The differential between other streptococci cannot be made on this picture alone, but in a very sick post-op or postpartum patient it is a rapid clue to suspect streptococcal or staphylococcal sepsis and a hint to undertake further sepsis workup and start timely antibiotic coverage. Small arrows: chains of cocci. Normal arrows: leukocytes indicating inflammatory response.

While on agar cultures *S. pyogenes* have a typical dome-shaped white-greyish appearance of >0.5 mm with a smooth or moist surface and clear margins, surrounded by a zone of β-hemolysis which is often two-to-four times as large as the colony diameter, microscopically *S. pyogenes* appears as Gram-positive cocci, arranged in chains (Figure 1). Wet mount has the advantage of being a rapid, cheap and easy to access bed side test [61]. It allows quick orientation in obstetrics, puerperal and post pelvic surgery patients to decide on further workup and the need for timely treatment.

*S. pyogenes* strains show the Lancefield group A antigen, but the presence of the group A antigen is not unique for *S. pyogenes*, as some *S. anginosus* group and *S. dysgalactiae* subsp. equisimilis isolates can also express these antigens [62]. To exclude these, bacitracin susceptibility (group A strep lacking β-hemolysis has lower susceptibility) or PYR determination tests (presence of the enzyme pyrolidonyl aminopeptidase) are needed. Rarely, non-hemolytic *S. pyogenes* may cause pharyngitis and invasive infections [63]. These strains may not be detected by current culture techniques. Modern labs use automated MALDI-TOF (matrix-assisted laser desorption/ionization time of flight) techniques, based on detection of the cellular proteome by mass spectrometry, to allow species identification as well as antibiotic resistance profiles [64].

##### Automated PCR Testing

Currently, numerous automated PCR-based techniques, allowing direct identification of bacterial pathogens from blood culture bottles or clinical throat samples, have been validated (e.g., Nanosphere Inc, Northbrook IL, USA.; BioFire Diagnostics LLC, Salt Lake City UT, USA; Hologic Inc., Marlborough MA, USA.) [61]. Importantly, point of care tests using this technology in throat swabs, such as the Cobas Strep A test (F. Hoffmann-La Roche AG, Basel, Switzerland.) and the Simplexa Group A Strep Direct Test (Focus Diagnostics, Inc., Lyndhurst NJ, USA) offer rapid PCR results for the detection of *S. pyogenes* for individual samples within 20 min, saving even more precious time to start adequate antibiotic treatment.

##### Antibiotic Resistance Testing

Until now, *S. pyogenes* remains uniformly susceptible to penicillin, making susceptibility testing superfluous, except in the case of penicillin allergy where other antibiotics need to be administered [65]. Susceptibility testing for macrolides should be performed using erythromycin, since resistance and susceptibility to azithromycin, clarithromycin, and dirithromycin can be predicted by testing against erythromycin.

##### Antigen Detection

“Rapid antigen tests” were developed for a group A-specific carbohydrate antigen in throat swabs by agglutination methods or immunoassays. Although these tests provide rapid results to enable initiation of antibiotics, conventional throat culture swabs remain the gold standard, as sensitivities of rapid antigen tests can be as low as 58% [66,67] and negative tests should be confirmed by a throat culture in children, but not in adults due to the lower of risk of streptococcal pharyngitis and rheumatic fever in this age group [68]. The specificity of rapid antigen tests is high, making a positive test reliable and secure enough to start treatment [69].

##### Serology and Serotyping

Serologic tests are mainly used to detect long term sequelae of GAS infections, such as rheumatic fever or glomerulonephritis, but these tests are not useful in acute infections. The most widely used antibodies for the diagnosis of poststreptococcal diseases are anti-streptolysin O and anti-DNase B.

Serotyping of GAS is based upon the antigenic specificity of surface-expressed T and M proteins. This information has no immediate diagnostic or therapeutic consequences and is not used in routine settings [70]. Recently, a molecular serotyping system has been established on the basis of the nucleotide sequence variations that encode the amino termini of M proteins, the emm gene [71]. Additional molecular typing techniques have been published [72].

## 2. Guidelines for GAS Genital Infection

### 2.1. Managing Infected Contacts

#### 2.1.1. General Prevalence of GAS Carriage

Pharyngitis due to *S. pyogenes* is mainly common in school-aged children. In a large meta-analysis, a pooled prevalence of 37% of children were found to have *S. pyogenes* as a cause of their sore throat [73]. In this review, the prevalence of GAS carriage among healthy children without pharyngitis was 12%, while other studies report that some 15–20% of asymptomatic school-aged children are colonized with GAS. Of asymptomatic household contacts of children with streptococcal pharyngitis, 25% also had GAS in their throat [74,75].

In a longitudinal follow up study of 100, 5–15-year-old asymptomatic children swabbed fortnightly in the throat, two or more sequential cultures positive for *S. pyogenes* were found in 27–32% of the cohort in each year of the study, corresponding to a 16% carrier rate per month (SD 4.99%; range 4.2–26%) [76]. Of these children, 53% were carriers of *S. pyogenes* at some point during their study.

In conclusion, we have to appreciate that (asymptomatic) children are a major reservoir of GAS in the population and can readily transmit the microorganism to their pregnant mothers or other contacts. Transmission occurs through droplet spread between children and adults, lasting for some weeks after acquisition. Once in a carrier state they have a lower density of *S. pyogenes* in their pharynx, decreasing the transmission risk [77]. Furthermore, the bacteria most likely to colonize individuals are assumed to be less virulent [24]. Amongst household contacts of 323 cases of iGAS, 12% had positive throat cultures within two weeks, with strains identical to the strain of the index patient. Of these, 33% had pharyngitis and none developed invasive disease. Young age and duration of contact with the index patient of 4 h or more were risk factors for colonization with *S. pyogenes*. The transmission risk of iGAS to other household contacts is only 0.3% [24].

#### 2.1.2. Eradication of the Carrier State

In general, antimicrobial therapy is not indicated for children with asymptomatic pharyngeal colonization [68]. Exceptionally, use of antibiotics can be defended if there is a family history of rheumatic fever or rheumatic heart disease (increased susceptibility), or during community outbreaks of *S. pyogenes* pharyngitis [78]. Eradication can successfully be achieved by a 10-day course of oral clindamycin [79] or, in an old study, by combining benzathine penicillin G and oral rifampin [80], but more readily by a 10-day course of amoxicillin plus clavulanic acid, or a first-generation oral cephalosporin [81].

#### 2.1.3. Risk Factors for *S. pyogenes* Carriage

Due to the large number of different M types, type-specific immunity is unlikely to prevent episodes of mild GAS disease or asymptomatic carriage [82]. As spontaneous eradication of *S. pyogenes* occurs in only half of patients [77,83,84], this implies that some children remain carriers over a long period of time [76]. Repeated environmental contact may also play a role. Emm types which are more likely to produce a biofilm [85] may allow *S. pyogenes* to evade host immunity and the action of antibiotics resulting in asymptomatic colonization. However, a longitudinal study did not demonstrate an association of *S. pyogenes* emm type with the likelihood of becoming a carrier [86].

Presence of PrtF1 and PrtF2 genes is related to adherence and internalization of the bacteria, leading to iGAS [87]. Isolates of prtF1 are 3 times more frequent in *S. pyogenes* carriers than in cases with bacterial eradication after antibiotic treatment [87,88,89,90,91]. Others found more prtF2, but not prtF1 genes in asymptomatic carriers than in pharyngitis cases [92]. Indeed, prtF2 positive isolates were shown to be more efficient in the internalization process [93]. Erythromycin-resistant isolates were more likely to be prtF1 positive and to invade cells more efficiently than erythromycin-susceptible isolates [94,95]. There is still considerable debate about the clinical importance of such findings as other virulence factors may play a much more important role.

GAS colonization is frequent in children everywhere in the world: 26% in Turkey [96], 16% in the US [76], 16% in Uganda [97]. In adults the carriage rate is much less and was 0% among 677 adults in Germany [98].

### 2.2. Incidental Finding of GAS Vulvovaginitis and Asymptomatic Vaginal GAS

GAS vulvovaginitis occurs sporadically in 2–7-year-old girls, but is rare in adult women, although exact data are lacking. Colonization occurs in 0.1–0.4% of asymptomatic women, and 2–3% of women with symptoms of vaginitis. If invasive GAS presents in adult women, most often it concerns puerperal infection or (pre-)sepsis. Its incidence is low, but mortality is high.

Streptococcal toxic shock syndrome (STSS) is also rare in adult women: but in Great Britain, in 49 TSS cases among children in 2008–2009, half were due to GAS [99]. This was similar to the low incidence rates in Minneapolis during the period 2000–2006 (0.5/100,000 inhabitants) and 0.7/100,000 in menstrual women [100].

### 2.3. Should We Screen for GAS and iGAS; If, Who, When and How?

#### 2.3.1. General Population

Screening for GAS means systematic searching in special regions of the body (vagina, throat, anus, or multiple) in all women (also men, children?) to identify and to eradicate GAS, which is potentially dangerous for the colonized person or surrounding individuals. Screening should have health benefits, no false-positive risks and should be cost effective in significantly reducing potential risks of the disease. Possible primary prophylaxis measures are: (1) prevent close contacts, (2) hand washing, (3) antibiotic treatment, (4) vaccines (in development).

#### 2.3.2. Children

Could or should we screen all children in kindergarten or school to prevent vulvovaginitis, scarlet fever, strep throat, etc.? This option is not feasible, nor desirable. Firstly, children with GAS pharyngitis have parents who also have a 12% probability of being carriers, so screening would need to be extended more widely. Second, the cost would be exorbitant. Finally, the number needed to screen and treat to prevent one case of iGAS is 271, which is far too high for practical instigation [101], and inappropriate when the global need for antibiotic husbandry is paramount.

#### 2.3.3. Pregnant Women

Due to the excess risks of GAS colonized women developing iGAS when compared to males (female/male ratio 4:1) [102] and the risk of iGAS in pregnancy being 20-fold higher than in non-pregnant women [52,102], it may look tempting to screen women at the beginning of pregnancy and treat them if positive. However, this would also pose cost–benefit issues [103]. Up to now, routine screening is not performed because the risk of preventing iGAS does not balance the burden of potential antibiotic treatment for both mother and child. Screening without treatment could be considered as a means of identifying potential at risk mothers. Further studies on the necessity and modalities of screening and treatment are needed.

#### 2.3.4. Screening Contacts of Patients with iGAS 

##### Family and Close Friends without a Risk Factor or Close Contact

A distinction has to be made between close contact (contact longer than 4 h) and household contacts (contact for 24 h in proximity during the last 7 days) [104]. In one study, after contact with a very severe fatal case of iGAS, a much higher proportion of household contacts (24 h/week) were infected (36%) than other contacts with less intense exposure (2%) [105]. However, the risk of transmission with invasive disease as a result was low (5/2874, 0.2%), with a NNT of 407 (95% CI: 273–807) for household contacts in good health (excluding couples age >75 and mother-neonate) and higher than the NNT for meningococcal meningitis 284 (95% CI: 156–1515) [101]. Therefore, routine screening for GAS and/or treatment and chemoprophylaxis are not recommended. Nevertheless, it is recommended that health care providers inform all household contacts about the early signs of potential GAS infection (localized muscle pain, sore throat, rise in temperature) and make them seek medical help as soon as this occurs, up to a period of 30 days after the index contact.

##### Household Contacts with a Risk Factor

For those household contacts at increased risk of developing iGAS in case of infection, chemoprophylaxis may be considered. The most pronounced risk factors are age (couples of 75 years or above) (NNT of 82 (95% CI: 46–417) and mother-neonate (NNT of 50 (95% CI: 27–393). Other risk factors such as HIV infection, IV drug use, chronic illnesses such as diabetes, heart disease and cancer, pregnancy and steroid use at age 45 years or higher are significant but less consistently associated in different studies [101]. Penicillin G (600.000 IE IM) + rifampicin (2 dd 250 mg) × 4 d, clindamycin (600–900 mg dd × 10 days) or azithromycin (250–500 mg dd × 5 days) can be used for this.

##### Sexual Contacts

There is a case report of ‘streptococcal sex syndrome’ developing cellulitis after sexual intercourse in a woman with deficient lymphatic pelvic circulation due to lymphadenectomy and irradiation [106], but no large or prospective data about the role of sexual transmission of GAS are available.

##### Health Care Workers

In six of eight surveys, a health care worker carried the same strain of GAS as the patient with iGAS under their care at delivery or surgery. Asymptomatic health care workers carrying GAS in nose, rectum or vagina have been the cause of outbreaks of invasive iGAS in hospitals and care centers for the elderly or disabled. Therefore, in any case of iGAS, increased awareness is warranted to supervise the potential infection of health care workers in close contact with patients, with a low threshold for performing cultures of throat, anus, skin lesions and vagina. If two or more cases of iGAS are noted in the unit within a 6-month period, there should be routine screening of all health care workers involved in care of the index patients before, during and after delivery and/or surgery. Chemo-prophylaxis is only initiated after finding positive cultures, and the health care worker should be suspended from work during the first 24 h of therapy.

#### 2.3.5. Patients with Previous iGAS

It is known that patients who recovered from iGAS have no proof of lifelong immunity, as there is evidence that some patients have had a second attack of iGAS some years after the first [107]. There are no specific guidelines on how to manage previous iGAS patients.

### 2.4. When Should We Be Alerted about Possible GAS Complications?

Lessons learned from serious case reports concern more rapid and systematic communication and dissemination of iGAS cases and mortality. Usually very detailed information about the circumstances wherein such cases develop is known, but often this information is not shared with all specialists.

Awareness of a high potential for transmission is paramount in young children aged 5–15 and their household contacts. If a pregnant woman is amongst the frequent or close contacts, selective screening by swabbing throat, vagina and/or anus of the pregnant mother could be considered.

If a pregnant woman is flagged by incidental positive findings of the presence of GAS (vide supra), there should be a low threshold for early antibiotic treatment if symptoms suggestive of potential iGAS develop.

During the postpartum period, very cautious follow up is warranted. (Pre-)septic women can often present with less obvious symptoms than generally expected. For instance, general malaise, extreme fatigue and only low-grade pyrexia can still indicate imminent sepsis and even a fatal outcome.

### 2.5. Treatment of GAS Infection

#### 2.5.1. Antibiotic Treatment

##### Asymptomatic Colonization

The finding of GAS in the throat or vagina of asymptomatic women does not generally necessitate treatment, unless there is a risk of spreading the micro-organism to people with an increased risk of invasive GAS disease, such as pulmonary patients, pregnant women, and elderly people with reduced health reserve. In such cases isolation and treatment with penicillin can be indicated.

##### Local Disease

GAS pharyngitis in children is usually self-limiting and only requires treatment if GAS is the proven cause and transmission to vulnerable people needs to be prevented. To prevent acute rheumatic fever, most physicians start treatment if the symptoms of pharyngitis are not gone after a week, as ARF occurs more often in those still symptomatic after 9 days. As guidelines may differ from country to country, and physicians often do not adhere to guidelines for strep pharyngitis in children, antibiotics are still often prescribed in symptomatic children [108]. Penicillin or amoxicillin are the drugs of choice, while cephalosporins or macrolides may be used in case of penicillin allergy. Impetigo does not always require antibiotic treatment but can be managed with gentle scrubbing with antibiotic or antiseptic soap. If treatment is deemed necessary, the potential involvement of *S aureus* and MRSA should be kept in mind, requiring use of clindamycin and linezolid until culture results are known.

##### Invasive GAS

As cannot be repeated enough, the first and crucial step is consideration of the disease and its recognition, even in the absence of typical symptoms of sepsis. In a critical patient, awareness and early recognition of imminent sepsis saves lives. After hemodynamic stability is achieved, early initiation of 2.4 million units of penicillin G 6 times daily and 600 mg clindamycin four times daily intravenously is indicated even when culture results are not yet known. Clindamycin is added in case of iGAS disease because, in septic patients, the expression of penicillin binding protein is jeopardized, and because clindamycin inhibits protein production in GAS bacteria leading to superantigens and M proteins.

#### 2.5.2. Antibiotic Resistance

In GAS organisms, unlike the closely-related *S. pneumonia*, resistance to penicillin is currently non-existent. In patients with allergy to penicillin, however, one has to take into account that 5–10% of GAS is resistant to macrolides [65]. If tolerated, therefore, cephalosporins are the preferred second choice in these patients. There is evidence that the proportion of macrolide resistance of GAS organisms is closely related to the (over-) consumption of these drugs in some populations, with China (Bejing), Poland, Italy and Spain having high resistance rates of 25% or more.

Due to high natural resistance, tetracyclines and fluoroquinolones are not suitable for use in patients infected with GAS.

#### 2.5.3. Hyperbaric Oxygen and Intravenous Immunoglobulins

According to some smaller studies the application of hyperbaric oxygen may be beneficial to heal acutely infected wounds. A recent Cochrane review, however, did not unequivocally confirm the benefit of such therapy in patients with invasive streptococcal disease [109,110]. Similarly, for treatment with intravenous hyperimmune globulins, contradictory reports have been published, and a recent Cochrane review did not show a clear benefit [110,111].

### 2.6. Prevention of GAS Infection and a Role for Vaccination?

#### 2.6.1. Prevention of iGAS

Historically, GAS was identified as the major cause of maternal morbidity and mortality from sepsis long before the identification of bacteria as a cause of infection and the notion that hand washing techniques could prevent the transmission of the disease. Since Semmelweis in 1847 provided compelling evidence of this, maternal outcomes have been improved by identifying risk factors and introducing hygienic measures such as hand disinfection of caregivers. Nowadays, hand washing with disinfectant soap and alcohol still remains the cornerstone of prevention, as transmission can occur directly from an asymptomatic colonized healthcare provider, other patients, or a community-acquired source. Training and education of perinatal intensive care unit teams to implement rapid and early diagnostic procedures and early intervention is also ongoing. However, despite this and the potential for prompt treatment of invasive GAS, it remains the major cause of severe maternal morbidity and mortality.

There are currently no guidelines for the prevention of transmission. It is also unclear how a patient with a previous history of GAS disease should be managed, and what the role could be for preventive strategies such as screening of pregnant women for GAS to prevent neonatal and maternal invasive GAS disease. Clinicians and scientists must work together to build national and international networks with the aim of developing a more complete evidence base for the treatment and prevention of these serious and devastating invasive infections, especially in underserved areas where the disease remains frequent and deadly. Therefore, screening studies are needed to evaluate baseline prevalence rates of GAS in the lower genital tract of pregnant women in antenatal clinics from different populations and geographic areas. Additional research is needed to further explore the sources of GAS, identify the specific invasive bacterial subtypes involved in severe invasive infections, and elaborate the pathogenesis of these pregnancy-related infections (e.g., superantigens) to generate novel preventative and therapeutic strategies. International societies like ISIDOG could play a key role in the establishment of research networks aiming to develop more evidence-based guidelines for clinical practice.

#### 2.6.2. Vaccination

Several obstacles hinder the development of a safe and efficacious vaccine for humans in the near future. Firstly, there is an enormous diversity of serotypes and antigens, which varies tremendously geographically. Secondly, there are serious worries about immunity development against GAS antigens which also enables auto-immune responses, as are known to occur in the form of acute rheumatic fever and glomerulonephritis after natural GAS infection.

Due to the latter risk, multivalent vaccines targeting the hypervariable N terminal region of the M protein, which is apparently not involved in the pathogenesis of auto-immune diseases, are currently being tested in humans. Still, although such vaccines may contain up to 30 antigens (30-valent vaccine), this will not cover all serotypes in the world, leaving South-east Asia and Africa out of scope, although the need for vaccines are largest there [112]. In order to overcome this huge antigenic variability worldwide, other vaccines aiming at the more conserved regions of some peptides are being studied, but only in animals thus far.

## 3. Conclusions

Severe GAS diseases in obstetrics (puerperal sepsis) or gynecology (vulvo-vaginitis, sepsis, STSS) are not frequent, but need fast, interdisciplinary and clinically experienced management.

The diagnosis is easy, if given by experienced clinicians.

Below are the summary and recommendations of the ISIDOG workgroup based on narrative literature review and expert consensus; grades of evidence are provided based on the Oxford Centre for Evidence-Based Medicine: Levels of Evidence (March 2019) accessible on: https://www.cebm.ox.ac.uk/resources/levels-of-evidence/oxford-centre-for-evidence-based-medicine-levels-of-evidence-march-2009, (accessed on 14 March 2021).

1. Timely Case detection

(a) Suspicion of iGAS should be high if:
  Post-operative and postpartum period (Grade 1b)
  Presence of GAS infection at ward or in staff (hospital acquired) (Grade 1b)
  Presence of sore throat, skin infection, purulent aerobic vaginitis, prior to admission (Grade 3b)
  Or close contact with such diseases (community acquired) (Grade 1b)
  Rapidly deteriorating patient (Grade 5)
(b) Investigations (Standard of care):
  Close follow up of vital signs and diuresis
  Cultures of blood, urine, wound, vagina
  Additional cultures of pharynx and anus for iGAS (Grade 2b)
  Infectious parameters (CRP, leukocytosis, lactate)
  Organ function tests (complete blood count, liver and kidney function)
  Imaging investigations (ultrasound, X-ray, CT-scan) depending on clinical presentation at the time.
(c) Notification:
  Even if no obligatory notification
  Try to find cause (patient, close contact, hospital contacts) (Grade 1b)

2. Management (Standard of care):

(a) Initial management: Don’t wait!
  Start IV penicillin or clindamycin before awaiting result
  Start 1L/1h of IV saline for initial shock resuscitation
  Multidisciplinary discussion when patient gets unexpectedly worse
(b) Patient isolation:
  Until 24 h after starting antibiotics—in case of wounds, until cultures are proven negative
  Disposable gowns, mask and gloves, daily cleaning and disinfection of room and material
  Instruct (and limit) visitors
(c) Cover any wounds with waterproof dressing
(d) Strict hand hygiene measures
(e) Inform household contacts about early symptoms and warning signs

3. Screening:

(a) No general screening of pregnant or pre-op patients (grade 1b)
(b) Treatment recommended in case of incidental finding in vaginal cultures/microscopy during pregnancy/(gyn) pre-op patients (grade 5)
(c) Source finding (grade 1b)
  Check for contacts of iGAS infected patients (including children with pharyngitis)
  If unexplained iGAS on ward, consider screening hospital staff involved and close contacts; also consider pathology and postmortem staff
  In case of an iGAS outbreak in hospital/care facility (>2): all health care workers with potential contact should be screened for GAS colonization
(d) Positive screening in health care workers:
  Eradication should be attempted by treatment 24 h off work

4. (Close) contacts of iGAS cases (Grade 1b):

(a) Screening (except for source finding) and chemoprophylaxis: NOT recommended (NNT is 423)
  Except mother-neonate pairs or couples >75 years of age
  During an outbreak (> 2 cases): lower threshold
(b) Monitoring:
  Close contacts and hospital workers should be clearly instructed in case of symptoms after risk contact with iGAS case to screen and seek treatment

## Figures and Tables

**Table 1:** GAS virulence mechanisms.

Mechanism of Action of *S. pyogenes*	Meditors and Their Activity
Attachment	M protein and lipoteichoic acid: mediating adhesion
	Pili (encoded by FCT genes (Fibronectin- and collagen binding proteins and T antigen encoding loci): adhesion to epithelial cells by forming biofilms
	Fibronectin binding protein (FBPs): extracellular matrix proteins
	Serum Opacity Factor (SOF): opacifies serum and binds to fibronectin
	Fbp54: mediates binding to specific human cells (e.g., buccal cells)
	Steptococal suface deghydrogenase (SDH): binds numerous host proteins such as laminin, plasminogen and fibronectin
	Streptococcal collagen-like proteins: mimics human collagen and increases binding to eukaryotic cells
	Hyaluronic acid capsule: prevents adherence to eukaryotic host cells
	Laminin binding proteins: interacts with SpeB protease to enhance attachment
	Streptococcal surface encolase (SEB): plasminogen binding protein
Evasion	Inhibition of host phagocytosis by an outer capsule of hyaluronic acid
	Streptodornase—a group of DNase enzymes which prevent killing by host neutrophils
	C5a peptidase—destroys a host chemotaxin (C5a), which is part of the complement cascade
	Streptococcal chemokine protease (ScpC)—an enzyme which degrades IL-8, preventing neutrophil ingress into tissue affected by necrotising fasciitis
Invasion	Exotoxins—Streptolysin S (cardiotoxic), Streptolysin O (haemolytic) and Streptococcal pyrogenic exotoxins (SpeA, SpeB, SpeE) which are responsible for TSLS
	Streptokinase—a protein (with subtypes SK1, SK2a & SK2b), which converts host plasminogen into plasmin, triggering fibrinolysis and thus tissue destruction

**Table 2:** Abbreviated Adult Case Definition for Streptococcal Toxic Shock Syndrome (CDC 2010).

Hypotension-Systolic BP <90 mm Hg AND Multi-organ Involvement Characterized by Two or More of the Following:
Renal impairment: Creatinine >177 µmol/L
Coagulopathy: Platelets <100 × 106/L or disseminated intravascular coagulation, defined by prolonged clotting times, low fibrinogen level, and the presence of fibrin degradation products
Liver involvement: Double the normal level of alanine aminotransferase, aspartate aminotransferase, or total bilirubin
Acute respiratory distress syndrome: defined by acute onset of diffuse pulmonary infiltrates and hypoxemia in the absence of cardiac failure or by evidence of diffuse capillary leak manifested by acute onset of generalized oedema, or pleural or peritoneal effusions with hypoalbuminemia
Skin involvement: a generalized erythematous macular rash which may desquamate
Soft-tissue necrosis, including necrotizing fasciitis or myositis, or gangrene

**Table 3:** Information on lab-based GAS prevalence in vaginal cultures from European countries provided by ISIDOG members.

Area/Country	Reporter	Total Number Vaginal Cultures	Vaginal GAS Positive	% GAS Positive
Switzerland (Basel)	B. Frey	22,762	73	0.10%
Switzerland (Geneva)	B. Martinez de Tejada	14,811	22	0.15%
Belgium (Tienen)	G. Donders	13,552	24	0.18%
Germany (Wupperthal)	W. Mendling	7767	29	0.37%
Sweden (Lund)	P-G. Larsson	3838	84	2.19% *
The Netherlands (Alkmaar)	A Adriaanse	21,995	531	2.41% *
**Total**		**84,725**	**763**	**0.9%**

* Samples only collected from symptomatic patients.

**Table 4:** Published prevalence of vaginal GAS colonization in asymptomatic pregnant women (panel a) and pregnant women with symptoms of vulvovaginitis (panel b).

Area/Country	*n*	Vaginal GAS	Status	Reference
(a) Prevalence in asymptomatic women				
US Northern New England	6944	0.03%	Normal pregnancy	[37]
Australia	1600	0.06%	Normal pregnancy	[38]
UK	100	0%	Normal pregnancy	[39]
The Netherlands	505	0%	Not pregnant, fertile age	[40]
(b) Prevalence in symptomatic women				
The Netherlands	505	4.9%	recurrent vaginal discharge	[40]
Pakistan	136	1.5%	Vulvovaginitis	[41]
Australia	50	6%	premenarchal girls > 2 years old with VV symptoms	[42]
Switzerland	80	21%	prepubertal girls, aged 2–12 years, with vulvovaginitis	[43]

**Table 5:** Risk factors of iGAS infection in an obstetrical setting.

1. Caesarean section vs. vaginal delivery (Grade 2 b) [51]
2. Location where labor and delivery occurred (developing country) (Grade 3a) [29]
3. Known GAS carriers (Grade 1b) [5,52]
4. Exposure to GAS carriers (Grade 1b) [45]
5. Altered immune status associated with pregnancy (20 × higher risk) (Grade 1b) [53,54]
6. Genetic background of the host (Grade 3a) [53,55]
7. Virulence of the infecting GAS strain (Grade 3a) [53,56]
8. Sore throat or upper respiratory tract infection (Grade 4) [53,57]
9. Retained placental products (Grade 2b) [53,58]
10. Purulent vaginitis (Aerobic Vaginitis) (Grade 5, see above Section 1.6.1)

**Table 6:** Clinical features of iGAS (pre-)sepsis in Ob/Gyn patients [2].

1. Fever
2. General myalgia
3. Tender, sub-involuted uterus
4. General malaise
5. Lower abdominal pain
6. Diarrhoea
7. Purulent and foul smelling lochia
8. Abnormal vaginal bleeding
9. Features of severe sepsis: hypothermia, tachypnoea, neutropenia
10. Signs of shock such as hypotension, and sustained tachycardia (sometimes absent)
11. Infection of other organs: wound infection (episiotomy, caesarian scar), mastitis, urinary tract infection, pneumonia, gastroenteritis, pharyngitis

## Data Availability

Data available at quoted references.
